# Dyskinesia estimation during activities of daily living using wearable motion sensors and deep recurrent networks

**DOI:** 10.1038/s41598-021-86705-1

**Published:** 2021-04-12

**Authors:** Murtadha D. Hssayeni, Joohi Jimenez-Shahed, Michelle A. Burack, Behnaz Ghoraani

**Affiliations:** 1 Department of Computer and Electrical Engineering and Computer Science, Florida Atlantic University, Boca Raton, FL 33431 USA; 2 Icahn School of Medicine at Mount Sinai, New York, NY USA; 3 Department of Neurology, University of Rochester Medical Center, Rochester, NY USA

**Keywords:** Movement disorders, Parkinson's disease, Neurological disorders

## Abstract

Levodopa-induced dyskinesias are abnormal involuntary movements experienced by the majority of persons with Parkinson’s disease (PwP) at some point over the course of the disease. Choreiform as the most common phenomenology of levodopa-induced dyskinesias can be managed by adjusting the dose/frequency of PD medication(s) based on a PwP’s motor fluctuations over a typical day. We developed a sensor-based assessment system to provide such information. We used movement data collected from the upper and lower extremities of 15 PwPs along with a deep recurrent model to estimate dyskinesia severity as they perform different activities of daily living (ADL). Subjects performed a variety of ADLs during a 4-h period while their dyskinesia severity was rated by the movement disorder experts. The estimated dyskinesia severity scores from our model correlated highly with the expert-rated scores (*r* = 0.87 (*p* < 0.001)), which was higher than the performance of linear regression that is commonly used for dyskinesia estimation (*r* = 0.81 (*p* < 0.001)). Our model provided consistent performance at different ADLs with minimum *r* = 0.70 (during walking) to maximum *r* = 0.84 (drinking) in comparison to linear regression with *r* = 0.00 (walking) to *r* = 0.76 (cutting food). These findings suggest that when our model is applied to at-home sensor data, it can provide an accurate picture of changes of dyskinesia severity facilitating effective medication adjustments.

## Introduction

Parkinson’s disease (PD), which affects over 6 million people globally^1^, is a progressive neurological disorder with motor and non-motor complications that impact the daily activities of persons with PD (PwP)^2^. PwPs at mid- and advanced-stages of the disease experience motor complications such as troubling motor fluctuations and dyskinesias^3,4^. Motor fluctuations from ON to OFF states are experienced as levodopa wears off between doses and the PD symptoms reappear^5^. Dyskinesias are abnormal, involuntary movements of different body parts that most often occur during the peak effect of levodopa in the medication ON state^6^. Although dystonic dyskinesias can also occur, choreiform is the most common phenomenology of peak dose levodopa-induced dyskinesia. At this stage of the disease, an accurate picture of changes in dyskinesia severity is needed to help the physician offer an effective medication adjustment^7^. Dyskinesia can be assessed using rating scales such as the Unified Dyskinesia Rating Scale (UDysRS^8^) and the modified Abnormal Involuntary Movement Scale (mAIMS^9^) amongst others^10^, but may not occur at their maximal severity when being assessed during an office visit, and so a clinical interview is often required. However, patient interviews are limited by patient recall, and clinical examinations may only provide a snapshot of dyskinesia, hence failing to capture an accurate picture of dyskinesia severity as it changes during patient’s medication states or in their natural living environment^11^. Our objective in this paper was to develop a sensor-based dyskinesia assessment system that can measure dyskinesia severity in the home-living environment while PwPs perform their normal activities of daily living (ADL). We postulate that such a system can objectively and continuously estimate dyskinesia to provide an accurate picture of changes in dyskinesia severity that is required for effective PD medication adjustments and enhanced care.

Technology-based dyskinesia assessment systems detect abnormal dyskinetic movements from the movement patterns captured using videos or inertial signals. Vision-based systems use pose estimation methods to estimate dyskinesia severity as PwPs perform some pre-defined tasks infront of a camera^12,13^. As a result, these systems can only provide intermittent estimation of dyskinesia instead of continuous estimations during ADL. Motion-based systems use inertial sensors to assess dyskinesia from the movement patterns. Rapid advancements in sensing technologies provide user-friendly wearables with a long battery life that can be worn by PwPs and used for dyskinesia estimation during ADLs without imposing significant restrictions^14,15^. Machine learning algorithms are used in these systems to detect the patterns of dyskinesia from the body movements data. Some of these algorithms focus on detecting the presence of dyskinesia, which is formulated as a classification problem^16–26^. However, for PD medication management, detecting the presence or absence of dyskinesia is not sufficient. The treating physician requires detailed information about the severity of dyskinesia at PwP’s daily living environment and to what extend it interferes with the PWP’s activities and impacts the quality of life^3,27^. Other algorithms estimate dyskinesia severity, which is formulated as a regression problem^28–34^. However, the main challenge in developing such algorithms is that short-term, dyskinesia-related movement patterns overlap with the patterns of the free body movements^35^. Therefore, most researchers have decided to estimate dyskinesia while patients perform some specific, pre-defined tasks such as resting and/or arm extended to eliminate the free-body-movement effect during. They use short-term, spectral-based features (e.g., power of 1–4 Hz band), and in combination with traditional machine learning methods such as linear regression or support vector machines, achieve a moderate to high correlation with the gold-standard dyskinesia scores measured by a neurologist during those pre-defined tasks^29,32,33^. In another work by Pulliam et al.^30^, both short-term spectral and temporal features are used in a linear regression model, which led to only a moderate correlation with the gold-standard dyskinesia scores during ADL. The limitation of these algorithms is that they require patients’ active engagement and provide only repeated snapshots (vs. continuous) measurements of the dyskinesia severity that can vary profoundly by the time of day. Also, they do not provide a high correlation with the gold-standard dyskinesia score when used during ADL^28^. Hence, these algorithms have limited value for PD therapeutic management^36^.

In this work, we developed a sensor-based assessment system based on two wearable inertial measurement units sensors placed on the upper and lower extremity of PwPs to estimate dyskinesia severity during ADL with a high correlation with the gold-standard dyskinesia score. We investigated the first application of advanced deep recurrent neural networks (RNN)^37^ to explore the long-term body movement patterns for dyskinesia estimation. Dyskinesia is characterized as short-term, erratic movements that interrupt the long-term patterns of ADL, thus we hypothesized that tracking the long-term temporal dependencies between the short-term spectral and temporal features during ADL can distinguish the dyskinetic erratic movements and significantly improve the estimation of dyskinesia severity. Long Short-Term Memory network (LSTM) is a type of RNN and has been successful in many applications (reviewed in^38^) including activity recognition using motion signals^39^. In this work, we used a bidirectional LSTM, which has the ability to capture both past and future long-term dependencies^40^. Our approach is novel for three main reasons. First, we developed the first dyskinesia severity estimation technique that truly considers both the long- and short-term patterns of body movements. Second, we provided a detailed analysis of the technique’s performance during different types of activities and medication states. Third, our approach provided an unprecedented performance for estimation of dyskinesia severity during ADL when compared to the existing approaches.

## Results

**Figure 1:**
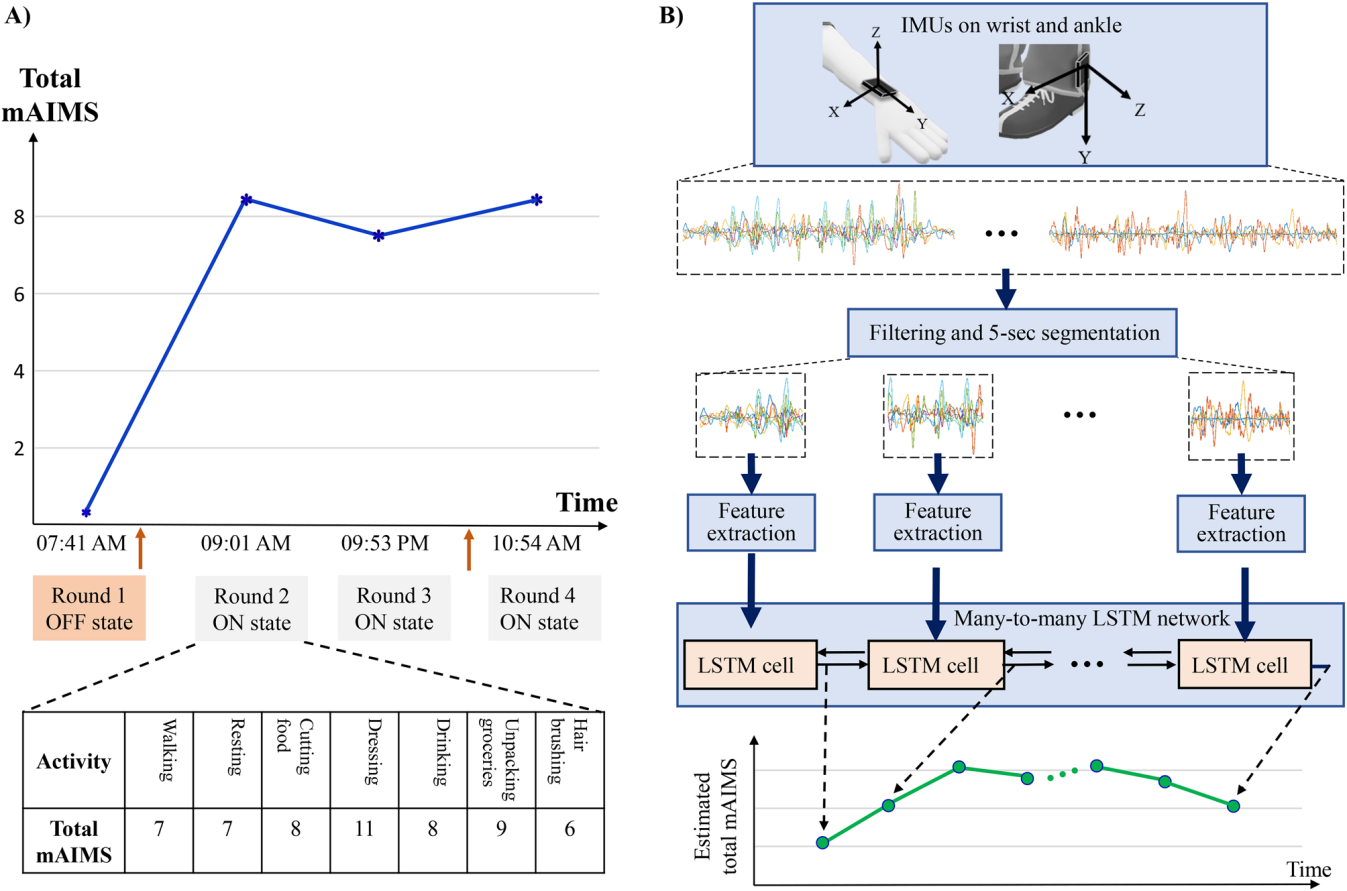
(**A**) The data collection protocol and timing of the four rounds of ADL as performed by one of the subjects. The list of ADLs and averaged mAIMS scores from the two raters are shown during different activities in round 2. The orange arrows indicate the timing of the PD medication intakes during the experiment. (**B**) A diagram representing the developed algorithm based on a bidirectional LSTM network. The input is the motion data from the wrist and ankle, and the output is the estimated dyskinesia severity score for every 5 s of the motion data.

We developed a new algorithm for the estimation of dyskinesia severity from movement data as PwPs performed different ADLs. The movement data was collected using one IMU (inertial measurement units) sensor placed on the wrist and one on the ankle of the most affected side of 15 PwPs. During a 4-h visit to the clinic, each subject engaged in four rounds of seven ADLs with about a 1-h gap between the rounds, except for two subjects who only performed three rounds. This process resulted in a total number of 58 rounds with each round being approximately 4-min long. Twelve of the subjects were in their OFF state during their first round. The activities were videotaped, and later dyskinesia was rated by two expert raters using mAIMS. The total mAIMS score ranged between 0 (no dyskinesia) and 28 (maximum dyskinesia). The average score of the two raters was used as the gold-standard dyskinesia severity score in this work. Figure 1A illustrates an example of the data collection protocol and the total mAIMS for one of the subjects. “Methods” provides the details of the data collection and experiment design. Figure 1B shows the developed machine learning algorithm and the details are provided in “Methods” section. First, the collected signals were filtered and segmented into 5-s windows. Next, a set of spectral and temporal features were extracted from each window. Finally, a bidirectional LSTM network was trained to associate a mAIMS score to each window. The developed algorithm was validated on the collected dataset in a leave-one-subject-out manner by training it on the data from 14 subjects and testing it on the remaining one, and repeating the process 15 times. For comparison purposes, we selected a linear regression model, as it has been commonly used by the research community for dyskinesia estimation, and trained and tested it on the same extracted features.

### Performance of the developed dyskinesia estimation model

**Figure 2:**
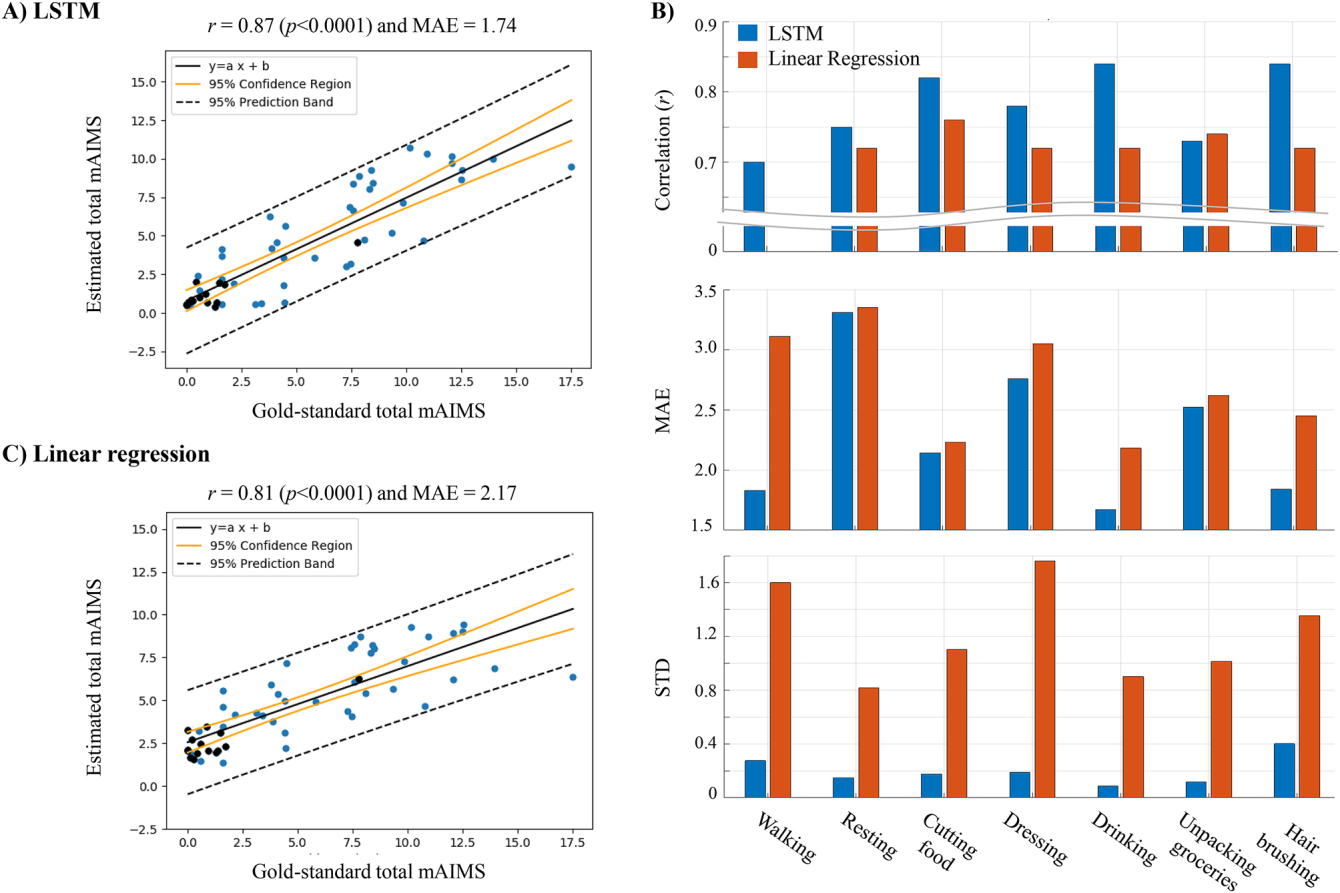
(**A**) The estimated scores from our LSTM model vs. the gold-standard mAIMS scores. (**B**) The average performance of the developed LSTM and linear regression that consists of the correlation, MAE and STD of estimated scores during different activities. (**C**) The estimated scores from the linear regression model vs. the gold-standard mAIMS scores. In (**A**,**C**), each data point represents one round of movement data. The black data points indicate the rounds recorded during the medication OFF state, and the blue data points represent the rounds in the medication ON state.

**Figure 3:**
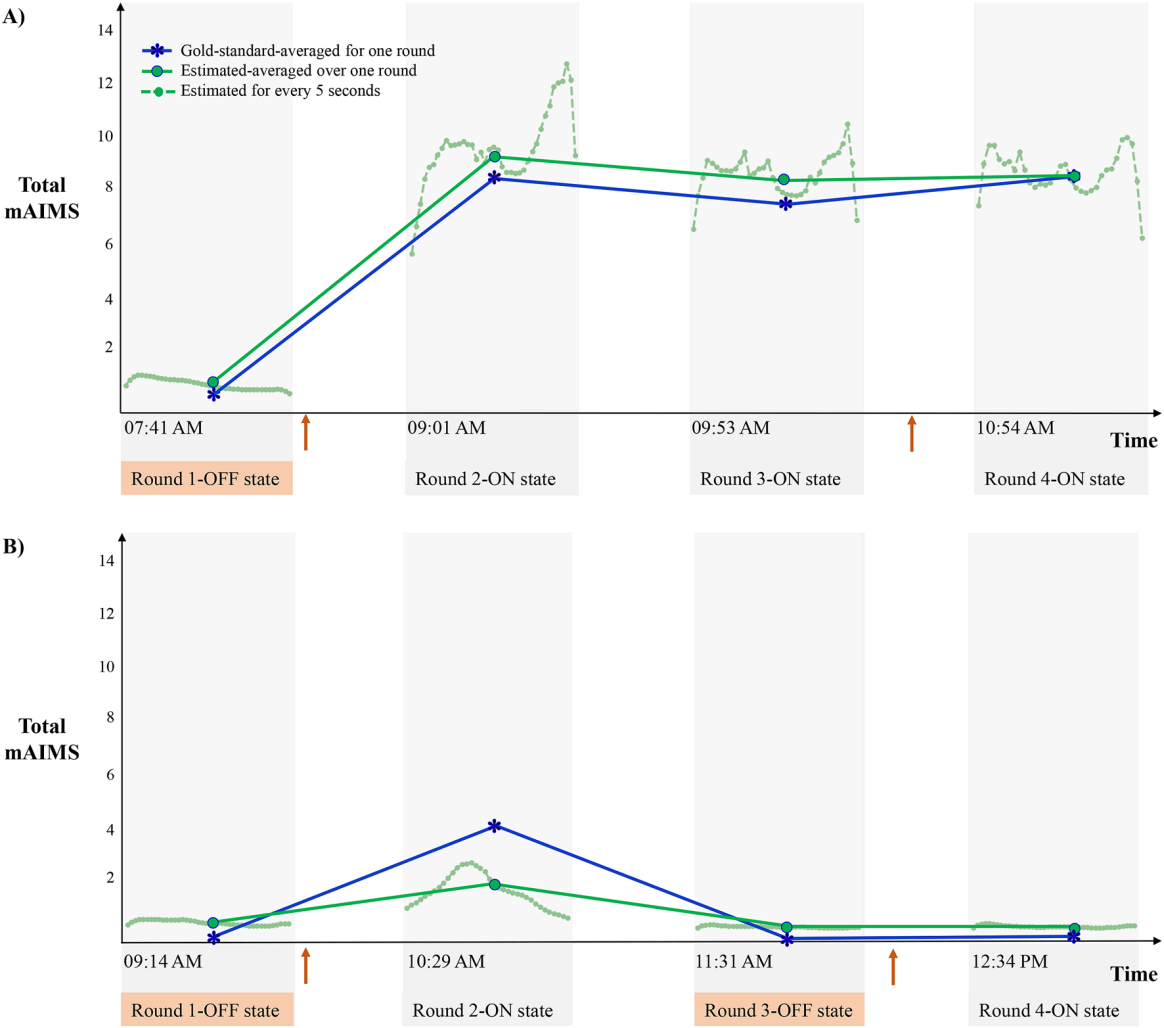
The estimated and gold-standard dyskinesia severity scores for two subjects (**A**,**B**). The estimated dyskinesia score over time by the LSTM model (solid-green lines) in comparison with the gold-standard mAIMS scores (solid-blue lines) averaged for each round of activities. The 5-s estimations of the dyskinesia scores are shown in the background in dotted lines. The beginning of each activity was shown only during round 2 of the first subject. The orange arrows indicate the PD medication intake time as prescribed by the treating physician.

Pearson correlation is widely used to evaluate the agreement between the estimated and clinical scores^28–32,34,41^ because a system with non-significant correlation will not be adoptable. However, the Pearson correlation by itself may not be sufficient to provide a comprehensive evaluation of the methods’ performance as it does not show the error between the two scores. That is why we used two metrics of Pearson correlation (*r*) and mean absolute error (MAE) between the estimated and gold-standard mAIMS scores for our evaluation purposes. We calculated the performance over 5-s windows and approximately 4-min rounds. First, we calculated the correlation r and MAE of the algorithm-estimated dyskinesia score over each 5-s window by comparing it against the gold-standard mAIMS score, which resulted in a correlation of *r* = 0.77 (p < 0.001) and MAE = 2.36 (8.4% of the maximum mAIMS score). It is a common practice to average the short-term, estimated scores of dyskinesia for a longer duration (2–30 min) to smooth the effect of any outliers in the estimated scores^28,33,34^. Therefore, we calculated the performance over rounds by averaging the 5-s estimations in each round and comparing it with the averaged gold-standard mAIMS scores. This process achieved a high correlation of *r* = 0.87 (p < 0.001) and MAE = 1.74 (6.2%). We also used the ranked-Spearman correlation for our evaluations. It resulted in a comparable correlation of r = 0.86, with only 1% lower than the Pearson correlation. Given that most of the existing literature on dyskinesia estimation use the Pearson correlation, we used the latter in the rest of work. The estimated dyskinesia scores over each round with respect to the gold-standard mAIMS scores are shown in Fig. 2A. Two examples of the LSTM model estimation of dyskinesia severity scores are shown in Fig. 3. These examples show the estimations at 5-s windows and 4-min rounds along with the gold-standard mAIMS scores provided by the expert raters. In the first example, the subject did not have motor fluctuations but had a total mAIMS of about 8 in the ON state. In the second example, the subject had motor fluctuations with mild dyskinesia. In both cases, the model estimated the dyskinesia severity closely when it was present and did not falsely report normal movements as dyskinesia when it was not present.

### Performance during different medication states

We investigated the performance of our model during medication OFF and ON states separately to ensure that the algorithm did not falsely estimate the PD symptoms such as tremor or bradykinesia as dyskinesia during OFF states. There were 16 rounds recorded during patients’ medication OFF state and 42 ON rounds. During the OFF state, there were only 5 rounds of ADL with a mAIMS higher than 1 (4 rounds with average mAIMS scores between 1 and 2, and one round with a 7.77 mAIMS score). The OFF rounds are shown as black data points in Fig. 2A. The model showed MAE = 0.75 (2.6%) during the OFF states and MAE = 2.12 (7.5%) during the ON state. The low MAE during the OFF state indicated that the model was not affected by the PD symptoms during this state, and at the same time it was able to estimate OFF dyskinesia accurately as shown in the black data points in Fig. 2A.

### Performance during different activities

The behavior of our dyskinesia estimation model during different types of activities was evaluated by comparing the correlation and MAE values during each of the seven ADLs that were performed in the protocol. We also calculated and compared the standard deviation (STD) of the estimated scores within a single activity as a metric to measure the sensitivity of our model to the short-term and erratic movements that may happen during voluntary body movements. In general, low STD values with a high correlation or low MAE during an ADL indicates the effectiveness and robustness of our model at dyskinesia estimation during that ADL. Figure 2B provides the correlation, MAE, and STD for the seven activities that were performed during the data collection protocol. Note that these results are based on the 5-s windows to provide sufficient granularity to separate performance for different activities. As Fig. 2B indicates, our model’s performance was relatively consistent during different activities with the STD values of less than or equal to 0.4. The model was not showing a rapid change in the estimated scores within activities, especially in the OFF states as shown in sample reports of Fig. 3. The correlation between the estimated and gold-standard dyskinesia scores ranged between 0.70 (for walking) and 0.84 (for drinking and hair brushing). The MAE ranged between 1.67 (5.9%) (for drinking) and 3.31 (11.8%) (for resting). The algorithm’s performance was relatively lower for walking and resting because walking and resting tremor movements have common temporal and spectral patterns with the dyskinetic movements, which make the dyskinesia estimation more challenging.

### The model sensitivity and specificity

**Figure 4:**
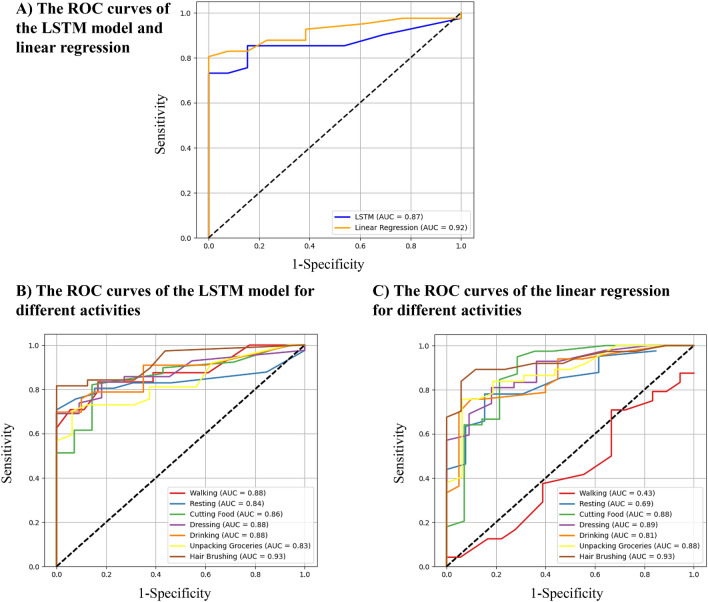
(**A**) The ROC curve for the LSTM model and linear regression considering the averaged estimated scores of all the activities in each 4-min round. (**B**,**C**) ROC curves for the LSTM model and linear regression, respectively, considering the estimated scores of a single activity each time.

We investigated dyskinesia detection using our LSTM model using a Receiver Operating Characteristic (ROC) curve. Each window or round of activities with a gold-standard mAIMS score of higher than or equal to 1 was considered positive. After thresholding the mAIMS scores, there were only 17 negative rounds and 41 positive rounds. We added our results as a new Fig. 4A. Figure 4A shows the ROC curve of the LSTM model with 0.87 area under the ROC curve (AUC). The best performance is 85% sensitivity and specificity using a threshold of 1.6. Figure 4B shows the LSTM ROC curves during different types of activities. The AUC was higher for all activities and ranged between 0.84 for resting and 0.93 for hair brushing.

### Feature analysis

We investigated the significance of the extracted features in estimating dyskinesia. For this purpose, we calculated the Pearson correlation between the numerical values of each individual features with the gold-standard mAIMS scores. The feature values were averaged across the axes of each sensor separately. This operation yielded only a single value for each one of the thirteen features for each round of activities. Using the wrist sensor, the peak to peak of the angular velocity had the highest correlation (*r* = 0.82 (p < 0.001)) followed by the standard deviation, power of secondary frequency, power of 1–4 Hz band, and Shannon entropy with *r* = 0.81, 0.80, 0.79, 0.78, respectively. Using the Ankle sensor, the second dominant frequency had the highest correlation (*r* = − 0.81 (p < 0.001)) followed by the Spectral entropy, Shannon entropy, Gini index, and standard deviation with *r* = − 0.80, 0.80, 0.80, 0.76, respectively. The correlation of the other features are provided in the Supplemental Table S1. The best features from the wrist sensor were different from the ankle sensor’s best features since the ankle and wrist were engaged differently in different ADLs. As a result, dyskinesia manifested differently on the wrist and ankle. More details about the correlation between the best features with the gold-standard mAIMS for different activities are included in the Supplemental Section S2 and Supplemental Figure S1.

### Comparison to the linear regression

We compared our developed LSTM model with a linear regression model with respect to the estimation accuracy as well as performance during medication ON and OFF states and different ADLs.

#### Accuracy

The performance of the 5-s estimations using a linear regression model was *r* = 0.64 (p < 0.001) and MAE = 2.85 (10.1%) and was increased to *r* = 0.81 (p < 0.001) and MAE = 2.17 (7.7%) after averaging the estimations over 4-min rounds. The estimated dyskinesia scores over each round with respect to the gold-standard mAIMS scores is shown in Fig. 2C. As hypothesized, the LSTM model was more successful in distinguishing the dyskinetic movements from ADL by modeling the long-term temporal dependencies between the short-term spectral and temporal features, which resulted in 20.31% (in *r*) and 17.19% (in MAE) improvement to the 5-s estimation performance and 7.41% (*r*) and 19.82% (MAE) to the 4-min estimation.

#### Medication ON and OFF states

The OFF state performance of the linear regression model was MAE = 1.67 (6%) and the ON state performance was MAE = 2.37 (8.5%). These results indicated that the LSTM model outperformed the regression model with 55.09% improvement during the OFF states and 10.55% during the ON states. As these numbers show and are also reflected in Fig. 2C, linear regression performed poorly during OFF states by estimating the dyskinesia severity of most rounds with a zero mAIMS score with around 2.5 dyskinesia score, indicating that it was impacted more by the symptomatic movements during the OFF states, while the LSTM model was more accurate in differentiating those patterns from the dyskinetic movements.

#### Different ADLs

The 5-s performance of the linear regression model with respect to different ADLs is illustrated in Fig. 2B. As the figure shows, the lowest correlation (0) was for walking, which was 0.70 using the LSTM model. The highest correlation (0.76) was for cutting food, which was lower than the highest correlation using the LSTM model (0.84) for drinking and hair brushing. The lowest and highest MAE values were found during drinking (2.18) and resting (3.35), respectively, which was similar to the LSTM model (1.67 for drinking and 3.31 for resting). The greatest difference in MAE between the two models was for walking where the LSTM model performed significantly better. The STD within a single activity of the estimated scores by linear regression was collectively higher than that of LSTM indicating that linear regression is more affected by the short-term voluntary movements mimicking erratic dyskinetic movements specially during dressing and walking.

#### The model sensitivity and specificity

The ROC curve of the linear regression model is shown in Fig. 4A. An AUC of 0.92 was achieved for this model. The best performance was 83% sensitivity and 92% specificity using a threshold of 3.4. When using the ROC analysis on skewed data (as in our case with more positives than negatives), a large change in the sensitivity can lead to only a minor change in the specificity and a better AUC^42^. Similarly, in our study with skewed data, the ROC analysis favored the linear regression model because it had a higher sensitivity. However, the AUC of the linear regression model showed lower performance than the LSTM model at the activity level. Figure 4C shows the ROC curves of the linear model during different ADL. The AUC of the linear regression model ranged between 0.43 and 0.93, while the LSTM model had an AUC of greater than 0.83 for all the activities. The AUC of walking and resting using the linear model were significantly lower than the AUC of the LSTM model.

## Discussion

PD is neither preventable nor curable. Only symptomatic managements exist, of which levodopa is the gold standard. Unfortunately, this treatment eventually leads to troubling motor complications, including fluctuations and dyskinesias. About 30% of PWPs develop dyskinesia within 5 years of levodopa treatment and increases to 90% when treating with levodopa for more than 9 years^43,44^. Effective dyskinesia management requires an accurate understanding of dyskinesia severity and duration over a typical day. To address this need, we developed a new algorithm to estimate dyskinesia severity during ADL with a high correlation with the gold-standard dyskinesia score from the movement data collected from one wearable, inertial measurement sensors placed on the upper and one on the lower extremity of the most affected side in PwPs.

Table 1 summarizes prior research towards estimation of the dyskinesia severity from body movement data, which have taken three main directions for algorithm development: *(i) Pre-defined tasks:* Most algorithms require the PwP to perform specific pre-defined tasks such as resting and extending the arms^29^, pronation and supination^32^, or sitting and standing still^33^. Among these algorithms, the work by Mera et al.^29^ reported the highest correlation of *r* = 0.85 (p < 0.001) when compared to the averaged mAIMS scores. *(ii) ADL:* Similar to our experiment, two algorithms employed the ADL performance. One algorithm resulted in *r* = 0.61 (p < 0.001) for an estimation duration of 30 s when compared to the leg dyskinesia item of the UDysRS score^31^ and the other one achieved *r* = 0.78 (p < 0.001) for a duration of 30 min when compared to the upper limb item of mAIMS score^34^. The former was based on linear regression and resulted in lower performance while the latter was based on convolutional neural networks and resulted in a better correlation. *(iii) ADL without walking:* The remaining two studies did not analyze walking during the evaluation. As showed by our analysis, a linear regression model results in a low correlation (*r* = 0 in our case) for dyskinesia estimation during walking. Hence, the low performance of linear regression could be the reason for reporting their results on ADL without walking with *r* = 0.80 (p < 0.001) for 12 min by Griffiths et al.^28^ and *r* = 0.77 (p < 0.001) for 12 s by Pulliam et al.^30^ when compared to the total mAIMS. Griffiths et al.^28^ reported only a weak correlation (p < 0.05) in the home environment.

We performed a series of experiments to investigate the performance of our developed LSTM model for estimating the dyskinesia severity ratings of mAIMS during different ADLs. Our first observation was that the LSTM model estimated dyskinesia with the highest correlation of *r* = 0.87 (p < 0.001) and a low estimation error of MAE = 1.74 (6.2%) with the total mAIMS in comparison with the existing algorithms based on the pre-defined tasks, ADL, or ADL without walking. As Table 1 shows most of these algorithms are based on a linear regression model, except for Pfister et al.^34^ which is based on convolutional neural networks. As shown in Figs. 2 and 4B,C, linear regression provides comparable but lower performance than LSTM for different ADLs because it lacks long-term connectivity in its formulation and cannot always differentiate between erratic body movements during dyskinesia and ADL. As shown in our results, linear regression results in a notably low performance during walking. The reason for such behavior is that the walking and dyskinetic movements share similar temporal and spectral patterns. The frequency of walking, which is 2 Hz on average^45^, overlaps with the frequency of dyskinetic movements, which is about 1 Hz–4^33^. As a result, without considering both the long- and short-term temporal and spectral patterns, a model is unable to estimate dyskinesia severity during most activities specially during walking. The developed LSTM model also outperformed the Pfister et al.’s^34^ deep learning approach based on convolutional neural network. Unlike LSTM, CNN models only the short-term patterns in the movement signals and cannot investigate the temporal relationships between those short-term movement behaviors. In this work, we used bidirectional LSTM which has the advantage over regular LSTM by utilizing the information from both past and future temporal patterns^40^.

**Table 1:** A list of existing methods for estimation of dyskinesia severity scores.

Reference	Method	PwP number	Sensor location	Site	PD state (ON, OFF)	Activity	Estimation interval	Gold-standard	Testing results
Griffiths et al.^28^	Linear regression	34	Wrist	Lab	–	ADL^a^	12 min	Total mAIMS	*r* = 0.80 (p< 0.001) Margin of error = 3.2
				Home	ON	ADL	2 min	UPDRS IV	Weak correlation (p < 0.05)
Mera et al.^29^	Multilayer neural network	15	Both wrists	Lab	ON$\backslash$OFF	Resting and Extending arms	20 s	Averaged mAIMS	*r* = 0.85 (p < 0.001) Mean-square-error = 0.3
Pulliam et al.^30^	Linear regression	15	Wrist and ankle	Lab	ON	ADL^b^	12 s	Total mAIMS	*r* = 0.77 (p < 0.001) Normalized root-mean-square-error = 9.4
Ramsperger et al.^31^	Linear regression	23	Ankle	Lab	ON	ADL	30 s	The leg dyskinesia item of UDysRS	*r* = 0.61 (p < 0.001) Margin of error = 1
		10		Home	ON$\backslash$OFF			–	–
Thomas et al.^32^	Linear regression, decision trees	19	Both wrists	Lab	ON $\backslash$ OFF	Pronation and supination	20 s	Treatment response scale^c^	*r* = 0.6 Root-mean- square-error = 1
Rodriguez et al.^33^	Single Feature	13	Waist	Home	ON$\backslash$OFF	Sitting or standing	30 min	UDysRS	*r* = 0.7 (p = 0.01)
Pfister et al.^34^	Convolutional neural network	30	Wrist	Home	ON	ADL	5 min	The upper limb item of AIMS	*r* = 0.75 (p < 0.001)
							30 min		*r* = 0.78 (p < 0.001)
The developed approach in this study	Bidirectional LSTM network	15	Wrist and ankle	Lab	ON$\backslash$OFF	ADL	5 s	Total mAIMS	*r* = 0.77 (p < 0.001) MAE=2.36
							4 min		*r* = 0.87 (p < 0.001) MAE = 1.74

^a^It was not mentioned if walking was included.

^b^Walking and resting were excluded from the ADL.

^c^A new scale introduced by Thomas et al.^32^

Our next observation was that the LSTM algorithm performed comparably well during both medication ON and OFF states with MAE = 1.67 (6%) and MAE = 2.37 (8.5%), respectively. The algorithm was able to estimate dyskinesia severity despite PwPs experiencing fluctuations between the medication ON and OFF states. As Fig. 3B shows the algorithm detected dyskinesia in round 2 when the PwP was in the ON state and estimated very low dyskinesia in round 1, 3, and 4 when the person was in the OFF state. This is important because it shows that the algorithm has the sensitivity to estimate dyskinesia during medication ON states where the dyskinesia is at its peak and during medication OFF state where PD symptoms (e.g., rigidity, bradykinesia, tremor) account for the kinematic changes rather than dyskinesia. However, some existing approaches in the literature were validated using data captured in the ON state only^28,30,31,34^, which achieved the highest performance of *r* = 0.78 (p < 0.001) for a duration of 30 min^34^. Other methods did not investigate their ON and OFF state performance separately. Our investigation indicates that linear regression generally performs lower than the LSTM model during OFF states and is affected by PD symptom-related movements, thus the MAE during the OFF state is higher (1.67 vs. 0.75 using LSTM) with many being falsely estimated at about 2.5 total mAIMS score (the black data points in Fig. 2C). It is also important to note that in addition to the peak-dose dyskinesia, some dyskinesia may occur during OFF states known as wearing off dyskinesia^10^. Hence, it is important for successful dyskinesia estimation algorithms to estimate OFF-state dyskinesia if present. Such an ability of our model can be observed by the close estimation of the dyskinesia scores during OFF rounds as indicated by the black data points in Fig. 2A.

Another important observation was the consistency of the LSTM performance during different ADLs. Our LSTM model was affected minimally by the normal movements in the ADL within a single activity (Fig. 2B—STD graph) or over different activities (Fig. 2B—correlation and MAE). This behavior can also be seen through the 5-s and 4-min estimations for two subjects in Fig. 3. The lowest performance was achieved during walking with *r* = 0.7 (p < 0.001), likely because the leg dyskinesia while walking was to some extent undermined by the voluntary movements of the legs. In comparison, the linear regression model was not able to successfully separate the dyskinetic movements from the voluntary movements, which resulted in *r* = 0 during walking and a high STD during dressing, walking, and hair brushing (Fig. 2B—STD graph). This behavior of the linear regression model explains the reason for most of the existing algorithms either restricting their estimations to some pre-defined tasks^29,32,33^ or excluding walking^28,30^ to improve performance.

This work used movement data from two sensors, one on the wrist and one on the ipsilateral ankle and reported an unprecedented performance with the total mAIMS score. Pulliam et al.^30^ also used the same sensor configurations to ensure that both upper and lower extremity dyskinesias are captured by the system. Other algorithms that used a single sensor on the wrist^28,34^ or on the ankle^31^, or one sensor on each wrist^32^ were validated against the dyskinesia score of that specific extremity. For example, Ramsperger et al.^31^ used one ankle sensor and achieved *r* = 0.61 (p < 0.001) for estimating only the leg dyskinesia item of UDysRS, or Pfister et al.^34^ used one wrist sensor and provided *r* = 0.78 (p<0.001) based on only the upper limb item of AIMS. Only the work by Griffiths et al.^28^ estimated the total mAIMS score using one wrist sensor and Rodriguez et al.^33^ estimated the total UDysRS score. However, the former excluded walking from ADL, and as a result achieved only a weak correlation (p < 0.05) when used in the home environment, and the latter was based on some pre-defined tasks.

Our algorithm reported a high performance of dyskinesia severity estimation based on the movement data collected from two wearable sensors, indicating the potential of our algorithm for objective and continuous estimation of dyskinesia severity during subjects’ routine daily life. Wearing two wearable sensors may be cumbersome for some PwPs; however, surveys have shown that PwPs have a good acceptance for wearing up to three sensors at home^46^. Additionally, dyskinesia manifests differently on different body regions as PwPs perform different activities^4^. Hence, investigation of both upper and lower extremity movements are required to provide a more comprehensive picture of dyskinesia severity as changes during free body movements. We evaluated our algorithm using data from 15 PwPs as they performed different ADLs over a 4-h clinic visit and reported high performance of dyskinesia severity estimation indicating the potential of our algorithm for objective and continuous estimation of dyskinesia severity. There were seven different ADLs in our experiment, which were comparable to the other papers that reported their ADL types^30,31^. However, performance of the algorithm in a larger cohort of PwP and in the home setting with potentially more complex activities warrants further investigations. Given that our dataset only provides the total dyskinesia score, investigating how dyskinesia manifests on different body regions during various activities warrants future work.

## Conclusion

A novel dyskinesia estimation approach was developed to continuously and objectively estimate dyskinesia severity scores as PwPs perform different ADLs. We developed the first algorithm, based on a bidirectional LSTM network, to consider the long-term temporal patterns of short-term spectral and temporal patterns of movement in estimating dyskinesia severity scores. For evaluation purposes, we used data from 15 PwPs who wore one IMU wearable sensor on the most affected wrist and one on the most affected ankle as they performed several ADLs during a 4-h clinic visit while their mAIMS dyskinesia scores were assessed by expert raters. Our comprehensive analysis of the algorithm and comparison with a linear regression model as well as the existing approaches in the literature validated our developed LSTM model for dyskinesia estimation in both medication OFF and ON states and during different ADLs. Our analysis demonstrated our algorithm’s high performance in estimating dyskinesia scores in the presence of free body voluntary movements that share common characteristic with dyskinesia. Our future work will extend the developed LSTM method to continuous home-based monitoring where the motion data are collected outside of a clinical setting.

## Methods

### Dataset

#### Participants

A study protocol was designed to collect motion signals using wearable sensors mounted on subjects with idiopathic PD^29,30^. The protocol was approved by the institutional review boards of the University of Rochester and Great Lakes NeuroTechnologies. Fifteen subjects who had a history of peak-dose dyskinesias participated in the study and signed an informed consent (6 F, 9 M; average age 58 ± 10 years; average disease duration 10 ± 4; total mAIMS 5 ± 4.4; average 26-item PD dyskinesia Scale (PDYS-26) 35 ± 21; and equivalent daily dose of levodopa 1226 ± 535).

#### Data collection protocol

Below we describe the experiment design and collection protocol for the movement and dyskinesia data^29,30^. All experiments were performed in accordance with the approved protocol.

##### Study design and movement data

 Inertial sensors (Great Lakes NeuroTechnologies Inc., Cleveland, OH) were used in the study to record the 3-dimensional angular velocity and acceleration with a sampling rate of 64 Hz. The subjects were asked to stop their PD medications the night before the experiment, so dyskinesia in both medication states can be observed and recorded. A the beginning of the experiment, two sensors were mounted on the wrist and ankle of the subject’s most affected side. Each subject performed four rounds of ADL as the motion signals were collected. The activities in each round were walking, resting by setting on a chair, using a knife and fork to cut food, putting on and taking off a coat, drinking water from a cup, unpacking groceries, and combing hair using the left then the right hand. After performing the first round, the subjects resumed their normal PD medications. A neurologist identified when the medication kicked in and then asked the subjects to perform the s round of experiment. The other two rounds were 1-h apart. The 4-h procedure allowed capturing the change in PD symptoms and dyskinesia during a long interval. Figure 1A shows the timings of the four rounds for one of the subjects. Nine subjects were due for another scheduled dose of levodopa before the fourth round. Each activity took between 15 to 60 s. Two subjects had only 3 rounds of activities because they started the experiment in the ON state, resulting in a total number of 58 rounds ($N_R$) for all the subjects. The recording time of all the rounds for one subject on average was 13.7 ± 1.6 min.

##### Dyskinesia assessment

Before each round, the Unified Parkinson’s Disease Rating Scale part III (UPDRS-III) was performed by a neurologist. Also, the experiments were video-taped. Later, the dyskinesia severity of each activity was rated by two movement disorder experts based on the recorded videos using the mAIMS score. The average of the total mAIMS scores from the two raters was used to minimize the effect of inter-rater variability, resulting in one gold-standard dyskinesia score for each activity trial. In mAIMS, a severity score between 0 and 4 (no dyskinesia to severe dyskinesia) is given for each one of the four extremities, head/neck, trunk and global. The total mAIMS is the sum of all these sub-scores resulting in a range between 0 and 28.

### Algorithm development

The collected gyroscope signals were passed through a 0.5–15 Hz bandpass filter to eliminate noise and gyroscope drift effect. The filtered signals were segmented, and multiple temporal and spectral features were extracted. Next, an algorithm based on a bidirectional LSTM network as shown in Fig. 1B was developed to estimate the dyskinesia severity score for a round of ADL. For comparison purposes, a linear regression model was implemented on the same extracted features.

#### Segmentation and feature extraction

We used a 5-s window without an overlap to segment the signals, which resulted in 2,280 windows. As Patel et al.^47^ has shown, a 5-s window is suitable to segment the motion signals prior to the extraction of dyskinesia features. Next, 13 temporal and spectral features were extracted from each axis. These features are listed in Table 2. Before extracting the spectral features, we first calculated the power spectral density for each window. The feature extraction process resulted in 13 features for each (*x*, *y*, *z*) axis, 39 for each wrist and ankle sensor, and a total number of 78 features ($N_f$) from each 5-s window. The calculation details and underlying justification of the extracted features are reported in Supplemental Section S1. In sum, a feature vector ($\mathbf {fv} \in \mathbb {R}^{N_f}$) was extracted from each 5-s window and provided a set of $\mathcal {D} = \{ (FV^{(r)},y^{(r)}) \}_r^{N_R}$ $(FV^{(r)} \in \mathbb {R}^{{N_{W}^{(r)}} \times N_f}$, $y^{(r)} \in \mathbb {R}^{{N_{W}^{(r)}}})$ where $FV^{(r)} = [\mathbf {fv}_1\mathbf {fv}_2 \ldots \mathbf {fv}_{N_{W}^{(r)}}]$, $N_{W}^{(r)}$ is the number of 5-s windows in round *r*, and $y^{(r)}$ is the gold-standard total mAIMS for each window in round *r* ($[y_1 y_2\ldots y_{N_{W}^{(r)}}]$). The extracted feature vectors and the associated mAIMS scores ($\mathcal {D}$) were next used to train and evaluate the LSTM or linear regression algorithms to estimate a dyskinesia score for every 5-s window.

**Table 2:** The extracted features.

Temporal features	Spectral features
Shannon entropy (#1)	Power of 0.5–15 Hz band (#1)
Gini index (#1)	Power of 1–4 Hz band (#1)
Standard deviation (#1)	Spectral entropy (#1)
Skewness (#1)	Dominant frequency and its power (#2)
Kurtosis (#1)	Second dominant frequency and its power (#2)
Peak to peak of the angular velocity (#1)	

The numbers indicate the number of extracted features from each axis.

#### LSTM

LSTM is an advanced type of deep RNN^37^ and has been used successfully in many applications that require the analysis of time series data^38^. LSTM consists of a memory cell and four sub-neural networks called gates which are input, modulation, forget, and output gates. These gates are connected in a specific architecture to efficiently learn the temporal dependencies in data^48^. Therefore, it is able to capture the dynamic, temporal patterns by tracking the long-term dependencies between the time samples. LSTM was shown in our previous work to be effective for the estimation of PD symptoms from free body movements^49^. In this work, we used a bidirectional many-to-many LSTM architecture to take advantage of both past and future states to accurately estimate dyskinesia severity scores^40^.

Training deep learning models on raw signals requires large datasets. However, most of the patient-based datasets such as the one in our study are relatively small for the application of LSTM on the raw movement signals. Hence, instead of feeding the raw movement signals, we used the extracted feature vectors *FV*. We fed the sequence of the extracted feature vectors from a round of ADL ($FV^{(r)}$) into the LSTM network and used a many-to-many LSTM design, as shown in Fig. 1B, to estimate a mAIMS score for each 5-s window in the 4-min round ($\hat{y}^{(r)}$). These 5-s dyskinesia estimations were then averaged to calculate the 4-min dyskinesia mAIMS score for each round.

#### Implementation

The LSTM network was implemented using Keras with TensorFlow backend^50^. The first layer of the network was a fully-connected layer that mapped the length of each feature vector to the number of LSTM hidden states ($N_h$). The layer used a ReLU activation function. Next, we used a bidirectional LSTM with $N_h$ hidden states and $N_l$ layers. The LSTM layer was followed by a fully-connected layer with a ReLU activation function and an output layer with a linear activation function. The performance was assessed using subject-based, leave-one-out cross-validation. To avoid LSTM from memorizing the order of the activities that were performed during the data collection experiment, we randomly shuffled the order of the activities in each round before training. This process will improve the generalizability of the trained model when applied to free body movements at a home environment. We added dropout layers after the fully-connected layers and between the LSTM layers. The Adam optimizer was used to train each network by minimizing the mean-squared-error loss for 150 epochs^51^. The learning rate was 0.001, and the batch size was 32 sequences. The dropout rate during training was 0.7. The hyper-parameters of the LSTM model were optimized using a validation set (20% of the training rounds). The optimized hyper-parameters were the number of layers from 1 to 3 and the number of hidden states from 64 to 320 with a step of 64. The network with the maximum validation *r* was used for testing in each cross-validation fold. The best LSTM network on the validation data had 3 layers and 128 hidden states.

#### Linear regression

The linear regression model was implemented using Scikit-learn library in Python. The model associated each extracted feature vector with its corresponding mAIMS score. The linear regression coefficients that were obtained during the training were used to weight the features of each testing feature vector to estimate the mAIMS dyskinesia score. The performance was evaluated using subject-based, leave-one-out cross-validation.

## Supplementary Information

Supplementary Information 1.
