# Supplementary material for: Dyskinesia estimation during activities of daily living using wearable motion sensors and deep recurrent networks
Source: Sci Rep. 2021 Apr 12;11:7865. doi: 10.1038/s41598-021-86705-1 (PMC8041801; doi:10.1038/s41598-021-86705-1)

Supplementary Online Content for "Dyskinesia Estimation During Activities of Daily Living Using Wearable Motion Sensors and Deep Recurrent Networks"

S1. Feature Extraction

We extracted thirteen temporal and spectral features from each axis. The total number of features from each 5-second window (W) was 78 since we were using two sensors, and each has three axes. The extracted features from the temporal domain are the following:

- The Shannon entropy captures the randomness in the time domain, and it is defined as follow:

$$H(W) = -\sum_{i=1}^{200} p(B_i) * \log p(B_i)$$

B_i represents one bin in a histogram bin set (B, range (−400, 400) with 200 steps) of W, and $p(B_i)$ is the probability of B_i. The range of the bin set (i.e. −400 to 400) represents the 75th and 25th percentile of the angular velocity readings.

- The Gini index captures the movement complexity in the time domain, and it is defined as follow:

$$G(W) = 1 - \sum_{i=1}^{200} p(B_i)^2$$

- The standard deviation:

$$\sigma(W) = \sqrt{\frac{1}{N}\sum_{i=1}^{N}(x_i - \mu(W))^2}$$

where N represents the number of samples in W which is (5*64), and $\mu(W)$ is the mean of W.
- The skewness:

$$S(W) = \frac{\frac{1}{N}\sum_{i=1}^{N}(x_i - \mu(W))^3}{\sigma(W)^3}$$

- The kurtosis:

$$S(W) = \frac{\frac{1}{N}\sum_{i=1}^{N}(x_i - \mu(W))^4}{\sigma(W)^4}$$

- The peak to peak of the angular velocity is calculated by calculating the difference between the mean of the highest 10 samples in W and the mean of the lowest 10 samples. Using the mean of 10 samples is to reduce the effect of outlies.

Before extracting the spectral features, we first applied N-point discrete Fourier transform:

$$F_k(W) = \sum_{n=0}^{N-1} W_n e^{-\frac{j2\pi}{N}kn}$$

Second, we calculated the power spectral density for each window ($PS(W) = |F(W)|^2$). The computed spectral features were:

- The power of 0.5-15Hz band is the summation of the powers of 0.5-15Hz band.
- The power of 1-4Hz band is the summation of the powers of 1-4Hz band. Dyskinesia usually occurs in this band.
- The spectral entropy is calculated by first dividing $PS(W)$ by the total power to normalize it then using Shannon entropy on each frequency point in the range 0.5-15Hz.
- The dominant frequency is the frequency associated with the maximum power in $PS(W)$.
- The power of the dominant frequency is the maximum power in $PS(W)$.
- The second dominant frequency is calculated by first finding the peaks in $PS(W)$ and specifying the minimum peak separation as 1Hz. Next, we find the frequency associated with the second highest peak.
- The power of the second dominant frequency is the second highest peak in $PS(W)$.

S2. Feature analysis

We investigated the significance of the extracted features in estimating dyskinesia. For this purpose, we calculated the Pearson correlation between the numerical values of each feature with the gold-standard mAIMS scores. The feature values of the 5-second windows were first averaged in each round of activities. Then they were averaged across the axes of each sensor separately. This operation yielded only a single value for each one of the thirteen features for each round of activities. Supplemental Table S1 shows the correlation coefficients between each feature and the gold-standard mAIMS scores for all rounds of activities using wrist and ankle sensors. We made several observations from this analysis. We found that the best features from the wrist sensor are different from the ones from the ankle sensor. The reason could be that wrist and ankle are engaged differently in different ADL, and as a result, dyskinesia manifests differently on the wrist and ankle. Using the wrist sensor, the peak to peak of the angular velocity had the highest correlation (r=0.82 ($p<0.001$)) followed by the standard deviation, power of secondary frequency, power of 1-4Hz band and Shannon entropy with r=0.81, 0.80, 0.79, 0.78, respectively. Using the Ankle sensor, the second dominant frequency had the highest correlation (r=-0.81 ($p<0.001$)) followed by the Spectral entropy, Shannon entropy, Gini index and standard deviation with r=-0.80, 0.80, 0.80, 0.76, respectively.

Supplemental Table S1 shows that some of the features have a very good correlation with the mAIMs scores if their values are averaged in each round of activities. However, in real situations at home, the patients usually spend more than a minute in each activity. Hence, we investigated the best features for each activity. The bar graph in Supplemental Figure S1 shows the correlation of these features for different activities. The individual features were not equally performing across activities. The best five ankle features had a low correlation to the mAIMS during walking,

but the wrist features had a better correlation during this activity. However, during resting and drinking, the wrist features had a moderate correlation, and the ankle features were performing better. The reason for the low performance during walking, resting, and drinking is that walking and drinking patterns and resting tremor movements have common temporal and spectral patterns with the dyskinetic movements. Using the feature from both sensors resulted in equal or better correlation than using a single sensor when fed to the LSTM model.

Supplemental Table S1: The Pearson correlation between each of the features using each sensor separately and the Gold-standard total mAIMS scores.

† indicates a non-significant correlation (p>0.05).

Wrist		Ankle	
Feature Name	*r*	Feature Name	*r*
Peak to peak of the angular velocity	0.82	Second dominant frequency	-0.81
Standard deviation	0.81	Spectral entropy	-0.80
Power of secondary frequency	0.80	Shannon entropy	0.80
Power of 1-4Hz band	0.79	Gini index	0.80
Shannon entropy	0.78	Standard deviation	0.76
Power of 0.5-15Hz band	0.77	Peak to peak of the angular velocity	0.76
Gini index	0.72	Dominant frequency	-0.72
Power of dominant frequency	0.65	Power of 1-4Hz band	0.68
Second dominant frequency	-0.62	Power of 0.5-15Hz band	0.67
Dominant frequency	-0.52	Power of secondary frequency	0.67
Skewness	0.26 †	Power of dominant frequency	0.59
Spectral entropy	-0.22 †	Kurtosis	0.27
Kurtosis	0.21 †	Skewness	-0.19 †

Supplemental Figure S1: The correlation between each of the five, best extracted features from each sensor and the gold-standard mAIMS scores for different activities.

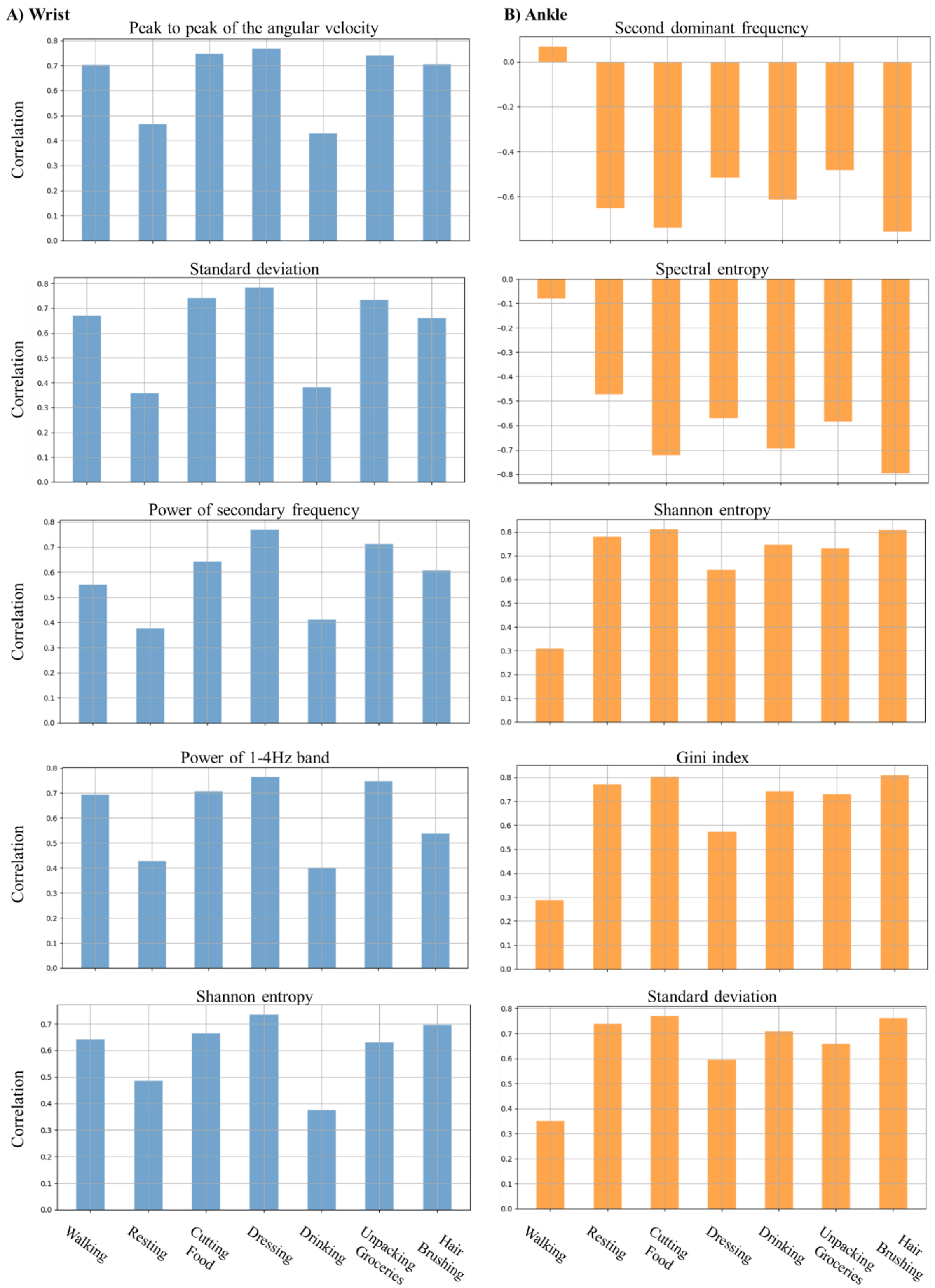

Supplement: Supplementary file 1 — Supplementary Information 1. [file 41598_2021_86705_MOESM1_ESM.pdf]
